# Supplementary material for: A transcriptome-wide association study identifies PALMD as a susceptibility gene for calcific aortic valve stenosis
Source: Nat Commun. 2018 Mar 7;9:988. doi: 10.1038/s41467-018-03260-6 (PMC5840407; doi:10.1038/s41467-018-03260-6)
Supplement: Supplementary file 1 — Supplementary Information [file 41467_2018_3260_MOESM1_ESM.pdf]

Supplementary Information

A transcriptome-wide association study identifies *PALMD* as a susceptibility gene for calcific aortic valve stenosis

Thériault et al.

Supplementary Table 1. Top GWAS results showing eight loci with at least one SNP with $P_{GWAS} < 1x10^{-6}$

SNP	Locus	Risk allele	Alt. allele	MAF cases	MAF controls	P_{GWAS}	Variant annotation	Closest gene	eQTL gene (P_{eQTL})*
rs116126498	4q32.2	T	C	0.041	0.017	1.35E-07	intergenic	*FSTL5*, *MIR4454*	None
rs954136	10q26.2	G	T	0.909	0.947	6.54E-07	intergenic	*DOCK1*, *NPS*	*MKI67* (0.045)
rs146580537	13q13.3	G	T	0.005	0.017	6.96E-07	intergenic	*FREM2*, *STOML3*	None
rs62280707	3q22.3	A	G	0.051	0.084	8.60E-07	intergenic	*FAIM*, *PIK3CB*, *CEP70*	*FAIM* (5.90E-05) *RBP2* (0.014)
rs62382996	5q23.2	G	A	0.110	0.068	2.39E-06	intronic	*LOC100505841*	*SNX24* (0.012)
rs141381550	14q12	A	G	0.010	0.028	2.42E-06	intergenic	*NUBPL*, *ARHGAP5-AS1*	None
rs148275869	4q22.2	A	C	0.037	0.016	2.56E-06	intergenic	*GRID2*, *ATOH1*	None
rs181762217	15q24.2	A	G	0.009	0.027	2.82E-06	intronic	*TMEM266*	*MIR631* (0.023) *GOLGA6C/D* (0.032)

*Valve eQTL-regulated gene (P_{eQTL})

Supplementary Table 2. Effect of the 12 SNPs included in the Mendelian Randomization instrument on *PALMD* gene expression and CAVS risk.

**rsID**	**Chr**	**BP**	**Effect Allele**	**Effect Allele Freq**	**beta eQTL**	**SE eQTL**	**P eQTL**	**beta GWAS Qc**	**SE GWAS Qc**	**P GWAS Qc**	**beta GWAS UKB**	**SE GWAS UKB**	**P GWAS UKB**
rs75131533	1	99931023	A	0.011	0.449	0.207	0.031	-0.748	0.331	0.020	0.051	0.198	0.796
rs115541361	1	99998481	T	0.046	0.228	0.077	0.003	0.079	0.161	0.622	-0.144	0.098	0.144
rs144406489	1	100001138	G	0.028	0.209	0.098	0.035	-0.262	0.217	0.226	-0.058	0.120	0.630
rs6702619	1	100046246	T	0.470	0.327	0.023	5.821E-33	-0.253	0.063	6.119E-05	-0.245	0.037	6.290E-11
rs72721723	1	100050140	G	0.010	0.478	0.179	0.008	-0.481	0.328	0.137	0.132	0.172	0.446
rs114323737	1	100065823	G	0.021	0.390	0.161	0.016	-0.200	0.242	0.406	0.039	0.115	0.732
rs115400609	1	100093163	G	0.021	0.239	0.102	0.019	-0.034	0.227	0.881	-0.127	0.115	0.271
rs77673766	1	100108802	G	0.011	0.570	0.206	0.006	0.027	0.322	0.932	-0.178	0.191	0.350
rs1416230	1	100133510	C	0.377	0.081	0.035	0.022	-0.016	0.066	0.802	0.023	0.038	0.544
rs1012097	1	100196610	T	0.104	0.135	0.054	0.014	0.033	0.104	0.751	-0.133	0.066	0.046
rs143673569	1	100266933	A	0.012	0.919	0.356	0.010	-0.191	0.472	0.686	-0.100	0.156	0.520
rs12121352	1	100276218	A	0.022	0.261	0.092	0.005	0.412	0.223	0.062	-0.118	0.129	0.362

CHR: chromosome, BP: position, Qc: QUEBEC-CAVS cohort, UKB: UK Biobank

Supplementary Table 3. Number of SNPs excluded at each quality control check.

**Quality control parameters**	**Total**
Call rate	22,856
Illumina GenCall	0
Hardy-Weinberg	139
Monomorphic or minor allele frequency	75,276
Different call rate in cases and controls	881
Total unique SNPs excluded	99,152
Total initial SNPs number	713,014
Total SNPs remaining	613,862

Supplementary Table 4. Number of samples excluded at each quality control check.

Quality control parameters	Total	Cases	Controls
Illumina GenCall	0	0	0
Call rate	8	5	3
Heterozygosity rate	1	1	0
Gender mismatch	0	0	0
Ethnicity	4	1	3
Duplicate and genetic relatedness	31	17	14
Total unique samples excluded	44	24	20
Total initial number of samples	2,070	1,033	1,037
Total samples remaining	2,026	1,009	1,017

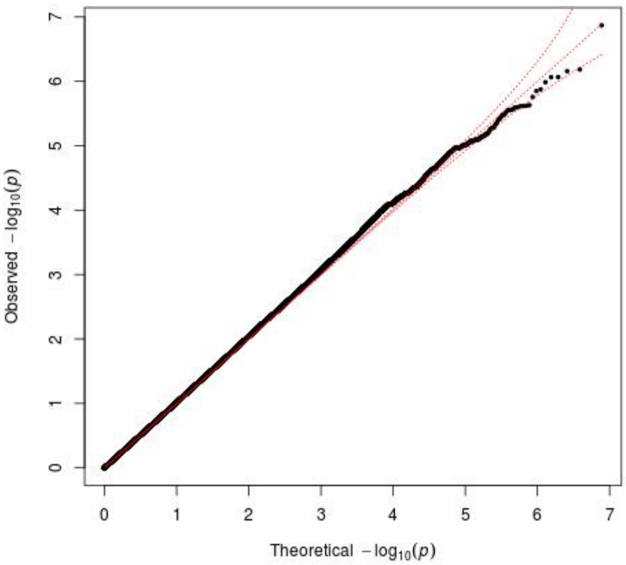

Supplementary Figure 1. Quantile-quantile plot of test statistics generated by the GWAS in 1,009 cases and 1,017 controls including 7,732,680 SNPs.

Supplementary Figure 2. Regional plots showing eight loci with at least one $P_{GWAS} < 1x10^{-6}$. The y axis shows the *P* values in -log10 scale for SNPs up- and downstream of the sentinel SNP (purple dot). The extent of linkage disequilibrium (LD; r^2 values) for all SNPs with the sentinel SNP is indicated by colors. The location of genes is shown at the bottom. SNPs are plotted based on their chromosomal position on build hg19.

a) 4q32.2

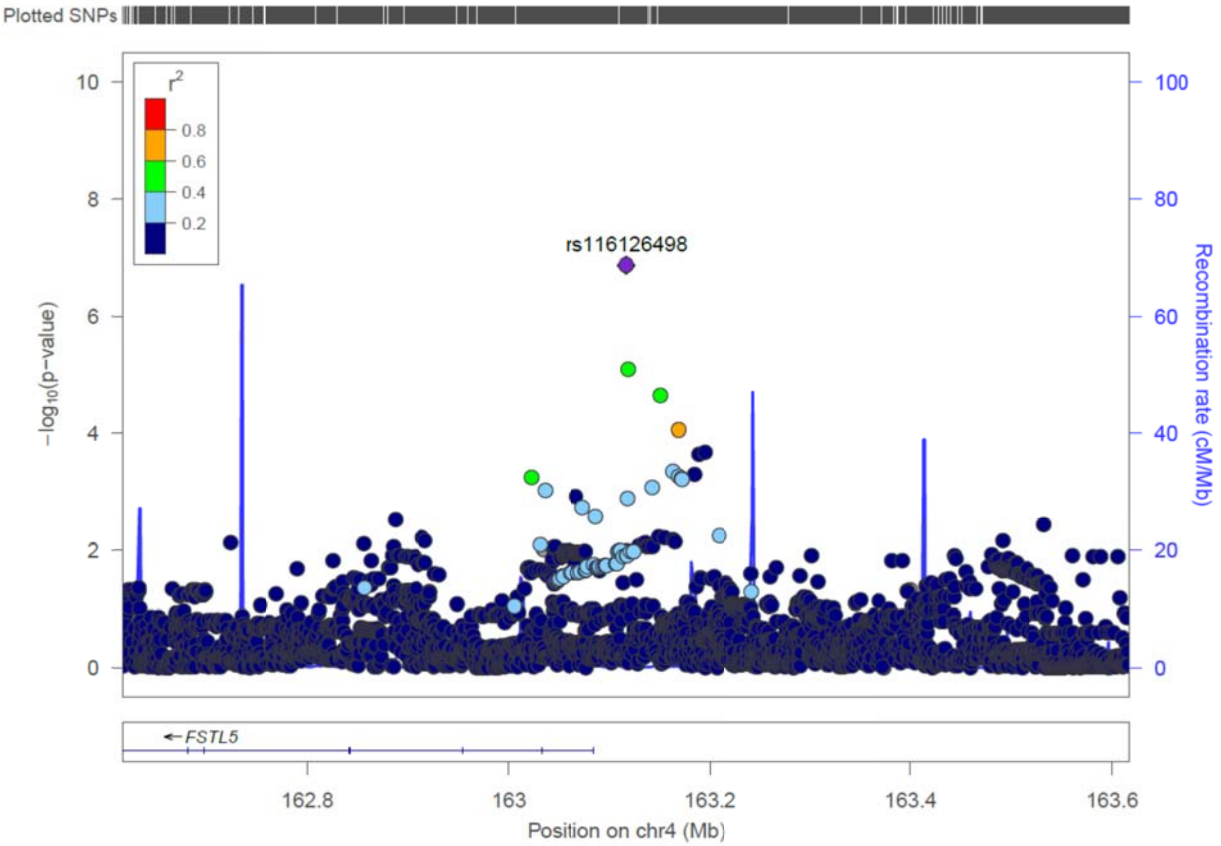

b) 10q26.2

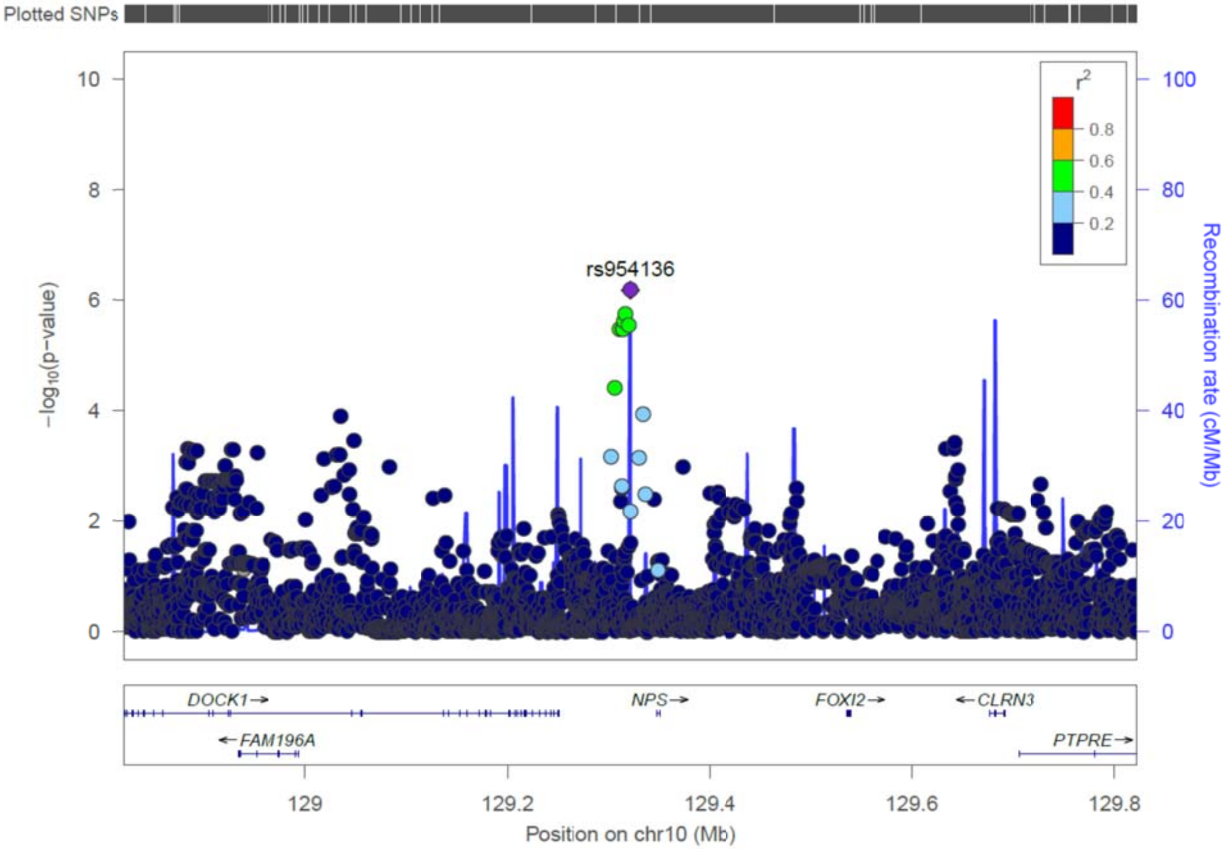
Plotted SNPs
rs954136
r2
0.8
0.6
0.4
0.2
−log10(p−value)
Recombination rate (cM/Mb)
DOCK1→
←FAM196A
NPS→
FOXI2→
←CLRN3
PTPRE→
129
129.2
129.4
129.6
129.8
Position on chr10 (Mb)

c) 13q13.3

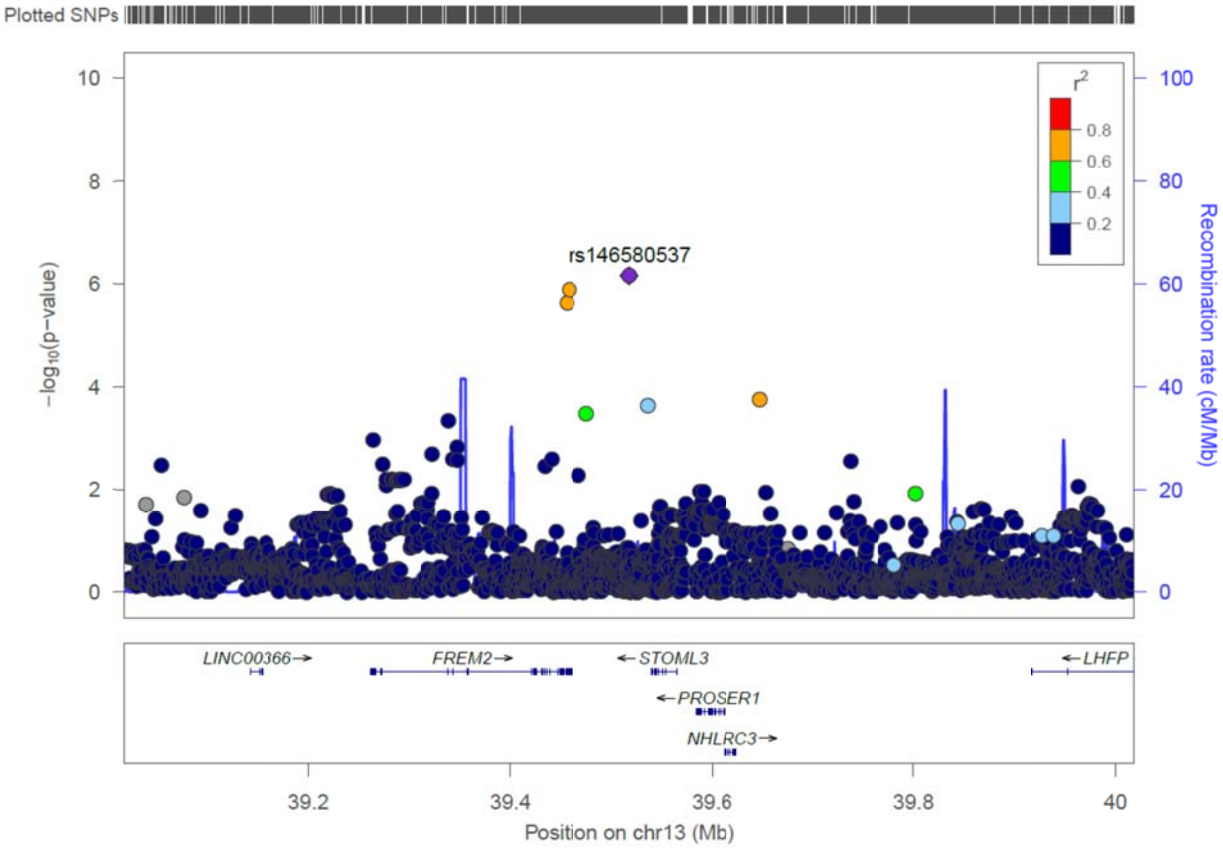
Plotted SNPs
rs146580537
r2
0.8
0.6
0.4
0.2
10
8
6
4
2
0
−log10(p−value)
100
80
60
40
20
0
Recombination rate (cM/Mb)
LINC00366→
FREM2→
←STOML3
←PROSER1
NHLRC3→
←LHFP
39.2
39.4
39.6
39.8
40
Position on chr13 (Mb)

d) 3q22.3

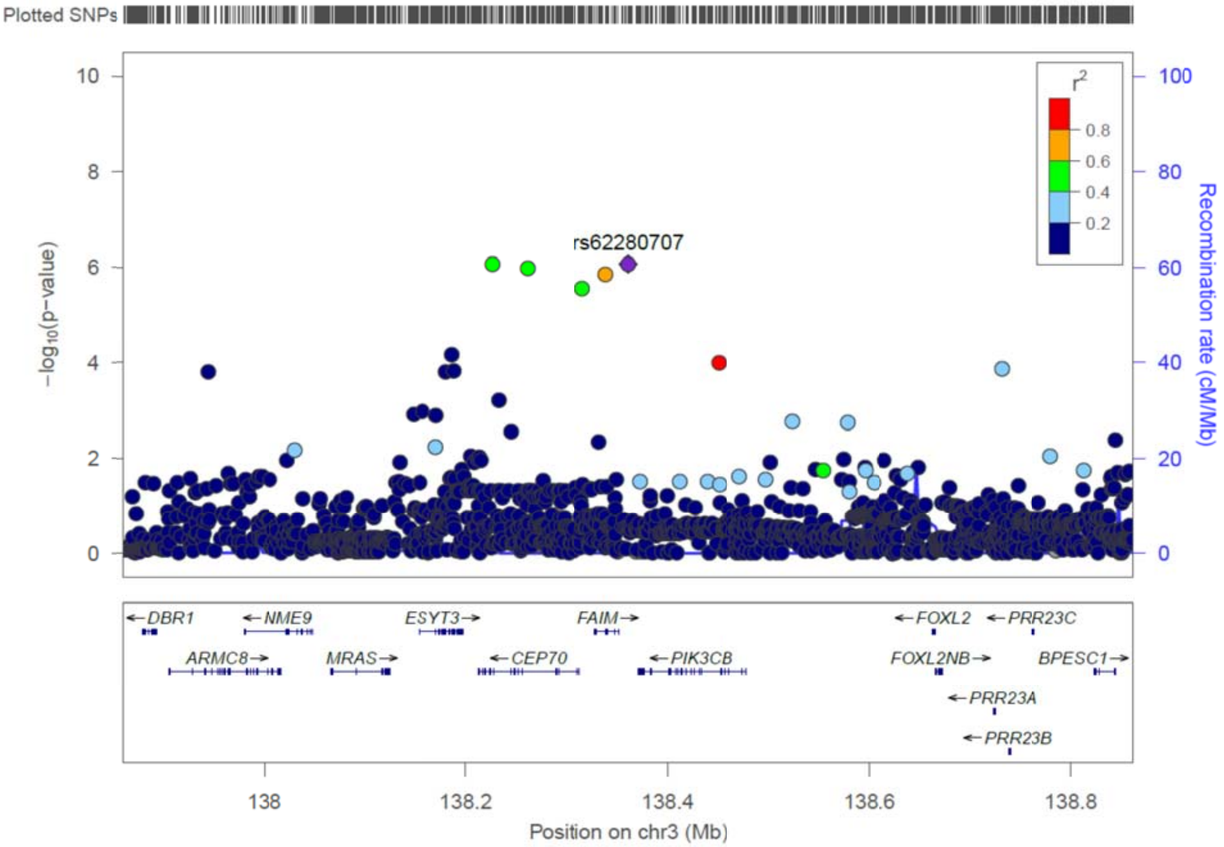
Plotted SNPs
−log10(p−value)
Recombination rate (cM/Mb)
rs62280707
r2
0.8
0.6
0.4
0.2
DBR1
NME9
ESYT3
FAIM
FOXL2
PRR23C
ARMC8
MRAS
CEP70
PIK3CB
FOXL2NB
BPESC1
PRR23A
PRR23B
138
138.2
138.4
138.6
138.8
Position on chr3 (Mb)

e) 5q23.2

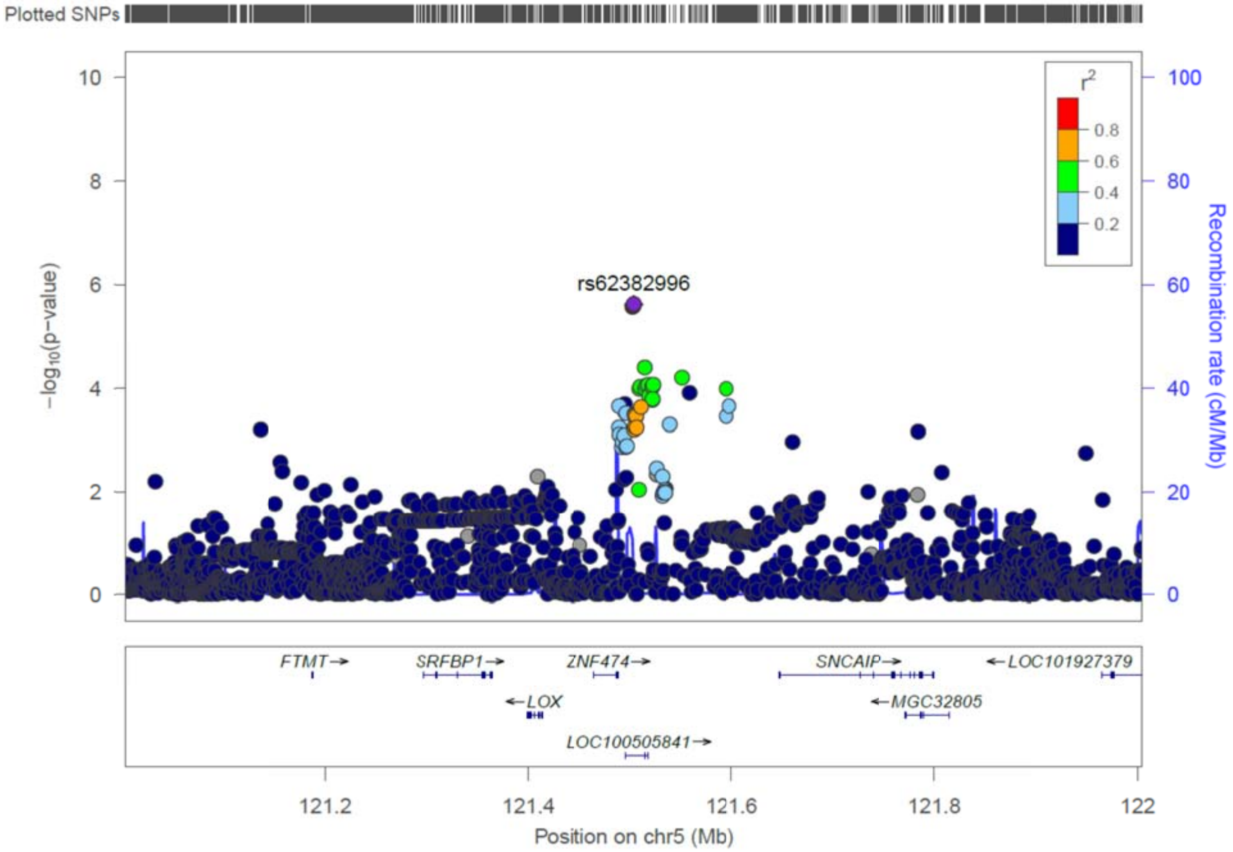
Plotted SNPs
rs62382996
−log10(p−value)
Recombination rate (cM/Mb)
r2
0.8
0.6
0.4
0.2
10
8
6
4
2
0
100
80
60
40
20
0
FTMT→
SRFBP1→
ZNF474→
SNCAIP→
←LOC101927379
←LOX
←MGC32805
LOC100505841→
121.2
121.4
121.6
121.8
122
Position on chr5 (Mb)

f) 14q12

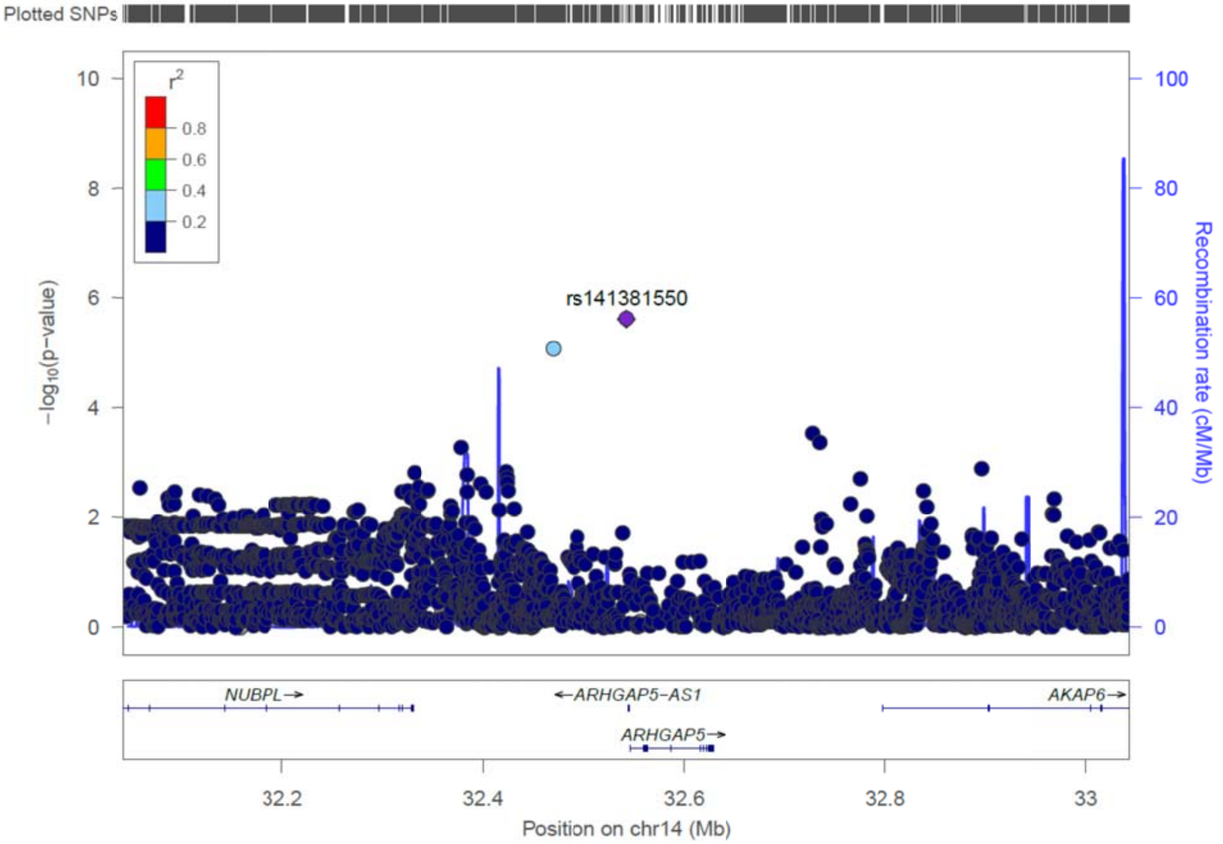

Plotted SNPs
10
8
6
4
2
0
r2
0.8
0.6
0.4
0.2
rs141381550
100
80
60
40
20
0
Recombination rate (cM/Mb)
−log10(p−value)
NUBPL→
←ARHGAP5−AS1
AKAP6→
ARHGAP5→
32.2
32.4
32.6
32.8
33
Position on chr14 (Mb)

g) 4q22.2

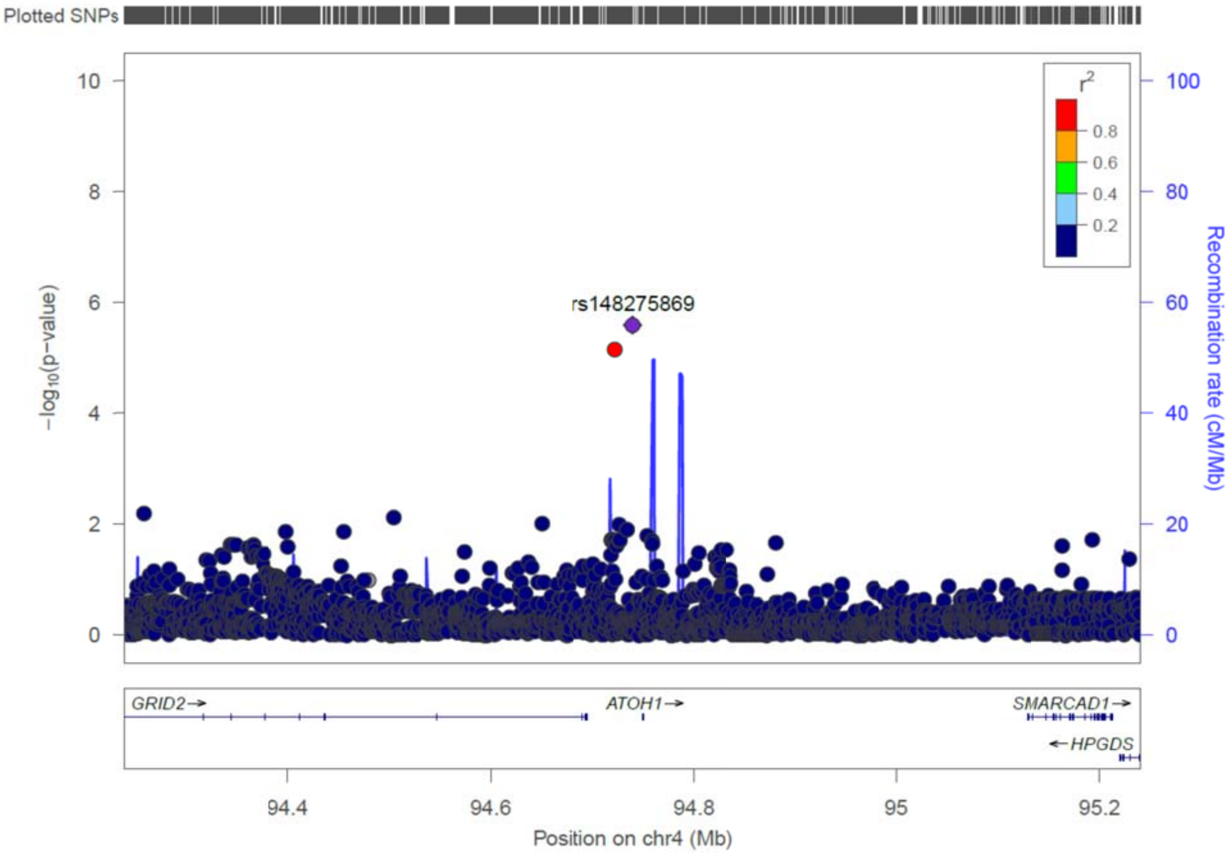
Plotted SNPs
10
8
6
4
2
0
−log10(p−value)
100
80
60
40
20
0
Recombination rate (cM/Mb)
r2
0.8
0.6
0.4
0.2
rs148275869
GRID2→
ATOH1→
SMARCAD1→
←HPGDS
94.4
94.6
94.8
95
95.2
Position on chr4 (Mb)

h) 15q24.2

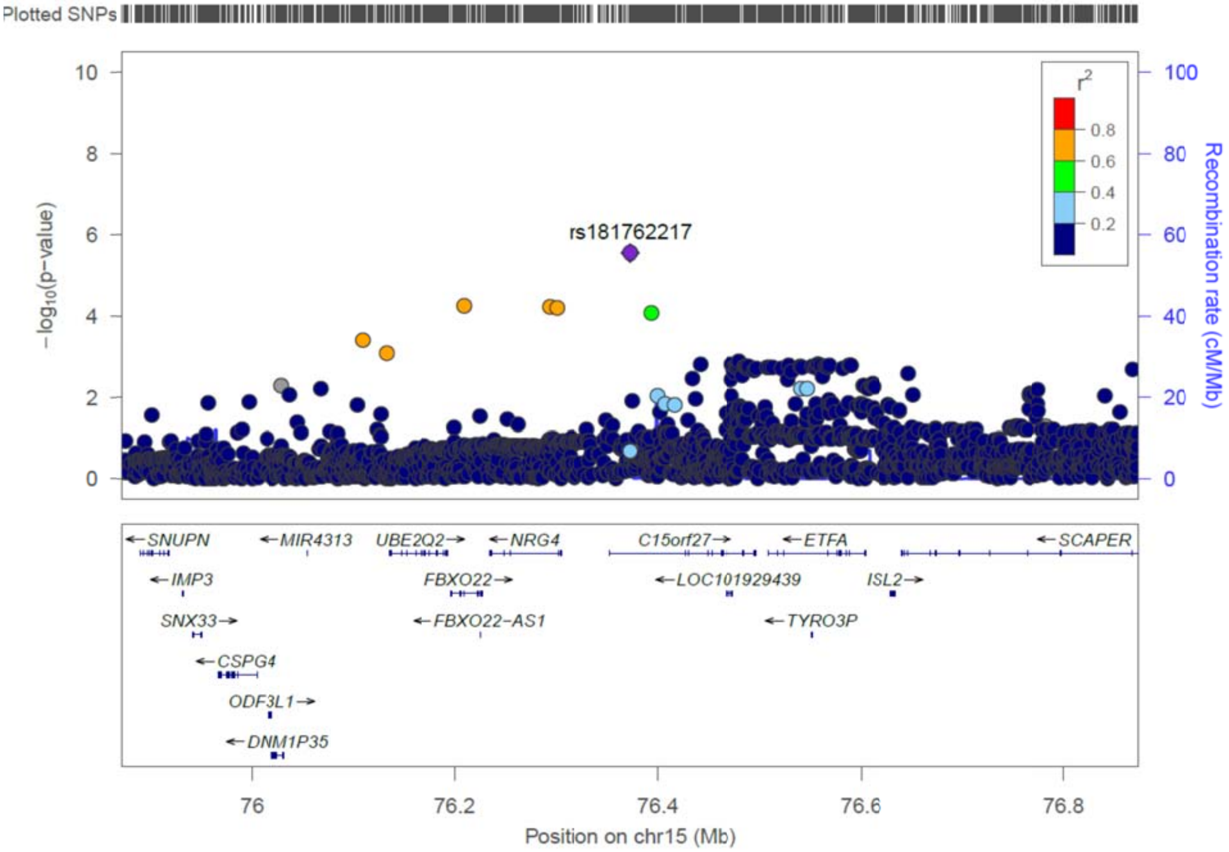
Plotted SNPs
10
8
6
4
2
0
−log10(p−value)
100
80
60
40
20
0
Recombination rate (cM/Mb)
r2
0.8
0.6
0.4
0.2
rs181762217
SNUPN
MIR4313
UBE2Q2
NRG4
C15orf27
ETFA
SCAPER
IMP3
FBXO22
LOC101929439
ISL2
SNX33
FBXO22-AS1
TYRO3P
CSPG4
ODF3L1
DNM1P35
76
76.2
76.4
76.6
76.8
Position on chr15 (Mb)

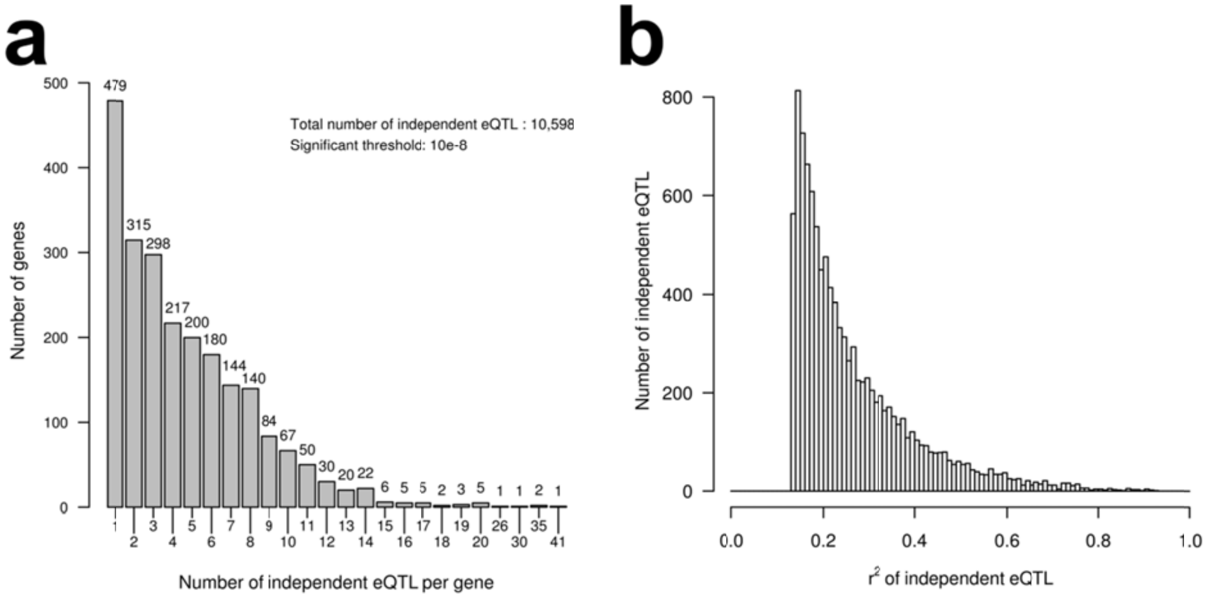

Supplementary Figure 3. Independent number and effect sizes of valve eQTL. (**a)** Number of independent eQTL identified. The y axis represents the number of genes/probes and the x axis is the number of independent eQTL per gene/probe, e.g. 479 genes/probes are significantly associated with one SNP; 315 genes/probes are significantly associated with two independent SNPs. (**b)** Distribution of effect sizes of SNPs on gene expression variance among the 10,598 significant and independent eQTL.

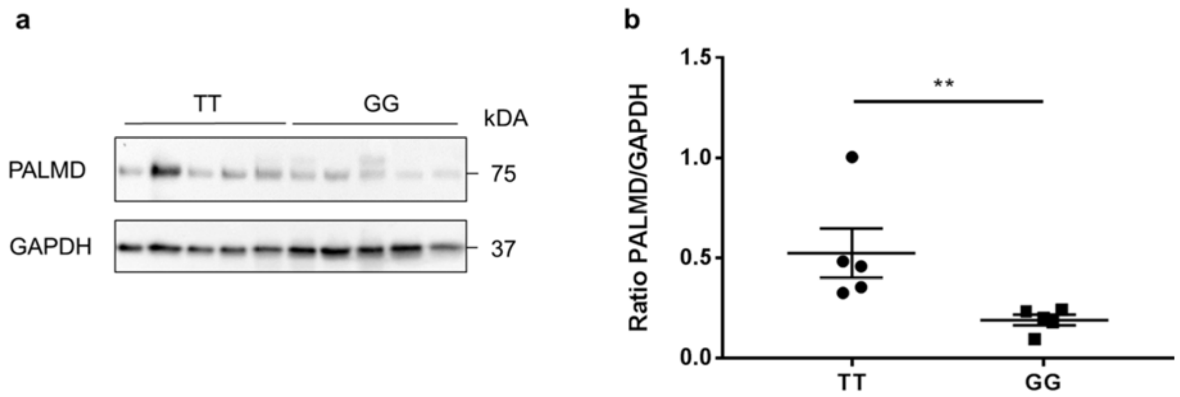

Supplementary Figure 4. Protein expression of PALMD by Western blot in human aortic valves. (**a**) Five homozygotes *TT* and five homozygotes *GG* for SNP rs6702619 were evaluated by Western blot normalized to expression of GAPDH. (**b**) The ratio PALMD/GAPDH is illustrated for each patient by genotyping groups. **p<0.01

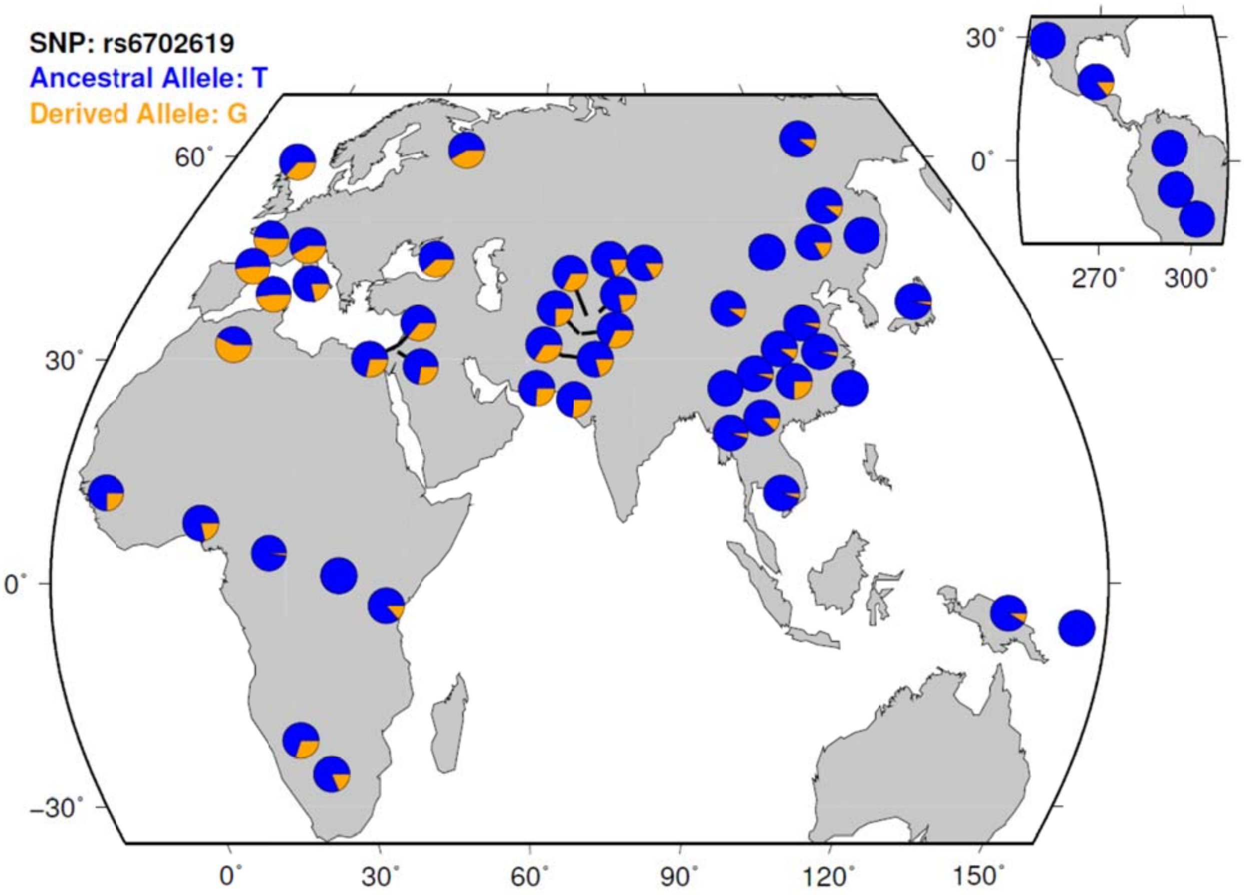

Supplemental Figure 5. rs6702619 global allele frequencies. The "G" (gold) allele at rs6702619 is present at higher frequency in European populations, as compared with the "T" (blue) allele. This figure was generated using the Human Genome Diversity Project Selection Browser[1].

References

1. Pickrell JK, *et al.* Signals of recent positive selection in a worldwide sample of human populations. *Genome Res* **19**, 826-837 (2009).
