# Supplementary material for: A transcriptome-wide association study identifies PALMD as a susceptibility gene for calcific aortic valve stenosis
Source: Nat Commun. 2018 Mar 7;9:988. doi: 10.1038/s41467-018-03260-6 (PMC5840407; doi:10.1038/s41467-018-03260-6)
Supplement: Supplementary file 3 — Description of Additional Supplementary Files [file 41467_2018_3260_MOESM3_ESM.pdf]

Description of Additional Supplementary Files

File Name: Supplementary Data 1
Description: The 10,598 independent aortic valve eQTL (PeQTL<1x10-8) identified in this study.
